# Pandemic (H1N1) 2009 among Quarantined Close Contacts, Beijing, People’s Republic of China

**DOI:** 10.3201/eid1710.101344

**Published:** 2011-10

**Authors:** Xinghuo Pang, Peng Yang, Shuang Li, Li Zhang, Lili Tian, Yang Li, Bo Liu, Yi Zhang, Baiwei Liu, Ruogang Huang, Xinyu Li, Quanyi Wang

**Affiliations:** Author affiliations: Beijing Center for Disease Prevention and Control, Beijing, People’s Republic of China;; Capital Medical University School of Public Health and Family Medicine, Beijing

**Keywords:** influenza, pandemic, close contact, attack rate, quarantine, viruses, China, pandemic (H1N1) 2009, research

## Abstract

We estimated the attack rate of pandemic (H1N1) 2009 and assessed risk factors for infection among close contacts quarantined in Beijing, People’s Republic of China. The first 613 confirmed cases detected between May 16 and September 15, 2009, were investigated; 7,099 close contacts were located and quarantined. The attack rate of confirmed infection in close contacts was 2.4% overall, ranging from 0.9% among aircraft passengers to >5% among household members. Risk factors for infection among close contacts were younger age, being a household member of an index case-patient, exposure during the index case-patient’s symptomatic phase, and longer exposure. Among close contacts with positive test results at the start of quarantine, 17.2% had subclinical infection. Having contact with a household member and younger age were the major risk factors for acquiring pandemic (H1N1) 2009 influenza virus infection. One person in 6 with confirmed pandemic (H1N1) 2009 was asymptomatic.

## Abstract 2

The attack rate was low, and having contact with an ill household member and younger age were the major risk factors.

In early April 2009, human cases of infection with a novel influenza virus of swine origin, pandemic (H1N1) 2009 virus, were identified in the United States and Mexico, and this virus spread rapidly across the world (*1**–**3*). On June 11, 2009, the World Health Organization raised the pandemic level to 6, the highest level for pandemic alert (*4*).

Estimating attack rates is a major task in characterizing pandemic (H1N1) 2009. Some studies have reported attack rates of pandemic (H1N1) 2009 among household members and aircraft passengers (*5**–**7*). These studies suggested that the transmissibility of pandemic (H1N1) 2009 virus was low. These studies were conducted in outbreak settings, and attack rates were calculated on the basis of clinical diseases that included influenza-like illness (ILI) or acute respiratory illness (ARI) of close contacts rather than confirmed infection with pandemic (H1N1) 2009 virus. In addition, in these studies only symptomatic index and secondary cases were included. Although most infections of pandemic (H1N1) 2009 influenza virus produce ILI or ARI symptoms (*8**–**12*), subclinical infection can occur and can change the estimate of attack rate. In addition, the infectivity of asymptomatic case-patients has not been clearly defined (*13*).

Because of the high rates of illness and death among the initial case-patients with pandemic (H1N1) 2009 (*14*), the Chinese government decided to prevent and contain the rapid spread of disease through tracing and quarantine of persons who had close contact with persons with confirmed cases of pandemic (H1N1) 2009. Beijing, the capital of the People’s Republic of China, took strict containment and control measures through October 2009. The Beijing municipal government implemented border entry screening, ILI screening in hospitals, health follow-up of travelers from overseas, and quarantine and testing of close contacts to identify new introduction of cases and local transmission. Public health workers conducted epidemiologic investigation of all index case-patients (including those with subclinical infections) and traced and quarantined close contacts whose residence was within the jurisdiction of Beijing. We estimated the attack rate of pandemic (H1N1) 2009 virus infection and assessed risk factors or correlates for infection among different types of close contacts, including household members and aircraft passengers.

## Methods

### Confirmation of Index Cases

In 2009, under the guidance of the Beijing Center for Disease Prevention and Control (Beijing CDC), a network of 55 collaborating laboratories was established to perform reverse transcription PCR testing to confirm cases of pandemic (H1N1) 2009 (*15*). The confirmed cases included symptomatic and asymptomatic cases, and these cases were detected mainly by border entry screening, ILI screening in hospitals, health follow-up of travelers from overseas, and quarantine and testing of close contacts. Once confirmed, index case-patients were immediately quarantined in designated hospitals to receive treatment while in isolation. All the confirmed cases were required by law to be reported to Beijing and local CDCs. From May through October 2009, a detailed epidemiologic investigation was conducted for each confirmed case of pandemic (H1N1) 2009 (including symptomatic and asymptomatic cases) by Beijing and local CDCs within 6 hours after confirmation of infection. Patients with confirmed cases were interviewed about demographic characteristics, course of illness, travel and contact history, and information about close contacts. Patients with confirmed cases were categorized as having imported cases (travelers) and locally acquired cases (no travel history) on the basis of where the infection was acquired.

### Definition of Close Contacts

Close contacts were defined as anyone who ever came within 2 meters of an index case-patient without the use of effective personal protective equipment (PPE) (including masks and gloves, with or without gowns or goggles) during the presumed infectious period. Trained staff from local CDCs made the determinations on the basis of field investigation. The relationships of close contacts to index case-patients were categorized as 1) spouses, 2) other household members, 3) nonrelated roommates, 4) contacts at workplace or school, 5) nonhousehold relatives, 6) passengers on the same flight, 7) friends, and 8) service persons met at public places. A close contact on an aircraft was defined as a passenger sitting within 3 rows in front and 3 rows behind the index case-patient.

All close contacts were traced and quarantined for 7 days after the most recent exposure to the index case-patient. All index case-patients detected between May 16 (the first case, the date of confirmation) and September 15, 2009 (before widespread transmission in Beijing), and their close contacts were included in this study. We excluded cluster or outbreak cases for which close contacts could not be determined clearly by epidemiologic investigation (the transmission chain was obscure).

### Laboratory Screening

For each close contact, before quarantine, a pharyngeal swab specimen was collected for reverse transcription PCR testing, regardless of symptoms. A second pharyngeal swab specimen was collected for testing for pandemic (H1N1) 2009 virus if any of the following symptoms developed in a close contact during quarantine: axillary temperature >37.3°C, cough, sore throat, nasal congestion, or rhinorrhea.

### Statistical Analysis

Data were analyzed by using SPSS version 11.5 (SPSS Inc., Chicago, IL, USA). Median and range values were calculated for continuous variables, and percentages were calculated for categorical variables. Differences in attack rates were compared between subgroups of close contacts by using the χ^2^ test. For the significant difference found in multiple subgroups, this test does not enable identification of which multiple subgroups are significantly different, only that across all the subgroups there are differences. The variables with p<0.10 in χ^2^ test were included in multivariate analysis. Multivariate unconditional logistic regression analysis was conducted to determine risk factors associated with infection in close contacts. Backward logistic regression was conducted by removing variables with p>0.10. Odds ratios (ORs) and 95% confidence intervals were calculated for potential risk factors of infection. The Hosmer-Lemeshow goodness-of-fit test was used to assess the model fit for logistic regression. All statistical tests were 2-sided, and significance was defined as p<0.05.

## Results

### Timeliness and Intensity of Index Case Detection and Contact Tracing

A total of 613 eligible index case-patients, detected from May 16 through September 15, 2009, were included in this study. Through field epidemiologic investigations, 7,099 close contacts were traced and quarantined in Beijing. The median number of close contacts per index case per day was 7.0 persons (range 2.0–95.0 persons); the median number for an imported index case was 7.0 persons (range 1.7–95.0 persons) and for a locally acquired index case was 5.3 persons (range 1.0–25.0 persons). For the 601 symptomatic index case-patients, the median interval between illness onset and sample collection was 1.0 days (range −1.9 to 7.0 days).

Among close contacts with symptomatic infection, the median interval between illness onset and sample collection was 0.5 days. More than 85% of close contacts were quarantined within 72 hours after interview of the index case-patients. The median interval between first exposure and quarantine was 3.4 days for the close contacts, and it was shorter, on average, for flight passenger contacts than nonpassenger contacts (1.7 days vs. 3.8 days). For symptomatic close contacts infected with pandemic (H1N1) 2009, the median of generation time (i.e., the time from illness onset in an index case to illness onset in a secondary case) were 2.4 days; it was shorter for flight passenger contacts than nonpassenger contacts (1.6 days vs. 2.5 days) (Table 1).

**Table 1:** Timeliness and intensity of contact tracing for pandemic (H1N1) 2009, Beijing, People’s Republic of China*

Contact type						Median no. days (range)			
	Hours from interview of index case-patient to quarantine of close contacts, % (no./total no.)†					First exposure to quarantine†	Last exposure to quarantine†	Illness onset to sample collection‡	Generation time‡§
	<24	>24–48	>48–72	>72					
All close contacts	41.3 (69/167)	26.3 (44/167)	21.6 (36/167)	10.8 (18/167)		3.4 (0.2–8.1)	1.9 (0.1–6.8)	0.5 (−4.5 to 5.0)	2.4 (0.2–6.8)
Flight passenger contacts	60.7 (17/28)	28.6 (8/28)	10.7 (3/28)	0 (0/28)		1.7 (0.2–5.6)	1.2 (0.2–3.8)	0.4 (−1.0 to 1.9)	1.6 (0.3–4.8)
Nonpassenger contacts	37.4 (52/139)	25.9 (36/139)	23.7 (33/139)	12.9 (18/139)		3.8 (0.2–8.1)	1.9 (0.1–6.8)	0.6 (−4.5 to 5.0)	2.5 (0.2–6.8)

*Data available for those who were infected because more detailed information was collected in field records. †Data for close contacts infected with pandemic (H1N1) 2009 influenza. ‡Data for symptomatic close contacts infected with pandemic (H1N1) 2009 influenza virus. Negative values indicate that the time of sample collection was before that of illness onset. §Generation time is time from illness onset in an index case-patient to illness onset in a secondary case-patient.

### Characteristics of Index Case-Patients and Close Contacts

Approximately 43% of the index case-patients were women; the median age was 20 years, and 38% likely contracted pandemic (H1N1) 2009 virus locally because they had not traveled recently. Among the index case-patientss, 2% had subclinical infection. Only 18% of index case-patients had close contacts with confirmed pandemic (H1N1) 2009 (Table 2), and the total number of close contacts who were infected by the virus from 110 index case-patients was 167.

**Table 2:** Characteristics of 613 index case-patients with pandemic (H1N1) 2009 and their close contacts, Beijing, China*

Characteristic	No. (%)
Index case-patients	
Female sex	265 (43.2)
Locally acquired cases	230 (37.5)
Subclinical infection	12 (2.0)
Infected close contacts	110 (17.9)
Close contacts, n = 7,099	
Female sex†	3,514 (50.0)
Relationship to index case-patient	
Spouse	75 (1.1)
Other household member	786 (11.1)
Nonrelated roommate	367 (5.2)
Contact at workplace or school	1,610 (22.7)
Nonhousehold relative	177 (2.5)
Passenger on the same flight	3,129 (44.1)
Friend	523 (7.4)
Service person met at public place	432 (6.1)
Contact phase	
Exposure to symptomatic index case-patient during symptomatic phase	4,305 (60.6)
Exposure to symptomatic index case-patient the day before illness onset	2,642 (37.2)
Exposure to person with subclinical index case	149 (2.1)
Type of quarantine	
Quarantine station	4,988 (70.3)
Home	2,111 (29.7)

*For index case-patients, median age (range) was 20 (1–75) y. For close contacts, median age (range) was 27 (0–99) y (data for 5,979 close contacts available). †Data for 7,032 close contacts available.

Fifty percent (3,514 of 7,032) of close contacts were women, and the median age was 27 years. Approximately 12% of close contacts were household member of index case-patients (spouse or other household member), and aircraft passengers accounted for 44% of close contacts. Approximately 61% of close contacts were exposed to symptomatic index case-patients during their symptomatic phase. About 70% were quarantined in a quarantine station (Table 2).

### Attack Rate

The overall attack rate for infection among close contacts (positive test result) was 2.4% (167 of 7,099), indicating that 1 index case-patient transmitted infection to 0.27 close contacts (167 of 613) on average (reproduction number = 0.27). Among those close contacts with a positive test result, 14.4% (24 of 167) had subclinical infection; among the close contacts with positive test results at the start of quarantine, 17.2% (20 of 116) had subclinical infection.

Attack rates did not differ by index case-patient’s sex (p = 0.225). However, attack rates differed significantly by index case-patient’s age (p = 0.022), and the lower attack rate was found for older index case-patients. There was no significant difference in attack rates between close contacts of patients with imported cases and those with locally acquired cases (p = 0.282). No infection was found in close contacts exposed to index case-patients with subclinical infection, and the attack rate observed in close contacts exposed to symptomatic index case-patients during their symptomatic phase was higher (p<0.001). Almost identical attack rates were found among male and female close contacts (p = 0.808). However, attack rates were significantly different among different age groups of close contacts (p<0.001), and the lowest attack rate was found for those >50 years of age. The attack rates were significantly different across 8 contact types (p<0.001). The attack rate was 5.3% among spouses and 6.6% among other family members in the household, and was lower among other types of close contacts (Table 3).The attack rate among passengers on the same flight was low, 0.9% overall, and 1 index case-patient transmitted infection to 0.19 close contacts on a flight on average (28 of 147), and the attack rate was higher among the passengers with longer flight times (>12 hours, p = 0.001). The attack rate among close contacts of service persons at public places was 0.2%, and 1 index case-patient transmitted infection to 0.01 close contacts of service persons on average (1 of 113). Nonpassenger close contacts with longer exposure duration (>12 hours), compared with those with shorter duration (>12 hours), recorded the higher attack rate (p<0.001) (Table 3).

**Table 3:** Attack rate for pandemic (H1N1) 2009, including subclinical infection, by characteristics of index case-patients and close contacts, Beijing, People’s Republic of China

Characteristic	Attack rate, % (no. infected/total contacts)	χ^2^ p value
Overall	2.4 (167/7,099)	NA*
Index case-patient		
Sex		
M	2.2 (91/4,192)	0.225
F	2.6 (76/2,907)	
Age, y		
0–19	2.7 (113/4,144)	0.022
20–50	1.9 (52/2,680)	
>50	0.7 (2/275)	
Infection source		
Imported case	2.5 (125/5,049)	0.282
Community-acquired case	2.0 (42/2,050)	
By type of exposure		
Exposure to symptomatic index case-patient in symptomatic phase	3.1 (135/4,305)	<0.001
Exposure to symptomatic index case-patient before illness onset	1.2 (32/2,642)	
Exposure to subclinical index case-patient	0 (0/149)	
Close contacts		
Sex		
M	2.3 (82/3,518)	0.808
F	2.4 (85/3,514)	
Age, y		
0–19	4.5 (82/1,837)	<0.001
20–50	2.4 (78/3,299)	
>50	0.8 (7/843)	
Relationship to index case-patient		
Spouse	5.3 (4/75)	<0.001
Other household member	6.6 (52/786)	
Nonrelated roommate	2.5 (9/367)	
Contact at workplace or school	3.0 (49/1,610)	
Nonhousehold relative	2.8 (5/177)	
Passenger on the same flight	0.9 (28/3,129)	
Friend	3.6 (19/523)	
Service person met at public place	0.2 (1/432)	
Flight time for passenger on the same flight, h		
<12	0.4 (8/1,846)	0.001
>12	1.6 (20/1,283)	
Exposure duration of nonpassenger close contact, h		
<12	1.9 (38/2,054)	<0.001
>12	5.3 (101/1,912)	

*Not available.

### Risk Factors

By multivariate analysis, age and type of contact were the major predictors of infection (Table 4). Compared with close contacts >50 years of age, those 20–50 years (OR 3.42; p = 0.002) and 0–19 years of age (OR 7.76; p< 0.001) were at higher risk for infection. Other significant independent risk factors associated with infection included being a household member of a person with an index case (OR 3.83; p<0.001), being exposed to index case-patients during their symptomatic phase (OR 1.86; p = 0.003), and exposure duration >12 hours (OR 1.83; p = 0.002). Similar risk factors were observed among aircraft passengers.

**Table 4:** Factors significantly associated with infection of pandemic (H1N1) 2009 virus in close contacts in multivariate analysis*

Factor	All close contacts†			Flight passenger contacts‡			Nonflight passenger contacts§	
	OR (95% CI)	p value		OR (95% CI)	p value		OR (95% CI)	p value
Age of close contacts, y								
>50	Reference			Reference			Reference	
20–50	3.42 (1.56–7.48)	0.002		3.13 (0.40–24.76)	0.280		2.89 (1.23–6.80)	0.015
0–19	7.76 (3.52–17.09)	<0.001		13.33 (1.77–100.22)	0.012		4.97 (2.06–12.00)	<0.001
Relationship to index case-patient								
Nonhousehold member	Reference			NA			Reference	
Household member	3.83 (2.65–5.53)	<0.001		NA	NA		2.37 (1.58–3.55)	<0.001
Type of exposure to index case-patient								
During asymptomatic phase¶	Reference			Reference			Reference	
During symptomatic phase	1.86 (1.23–2.80)	0.003		NA	NA		1.79 (1.09–2.93)	0.021
Exposure duration of close contacts, h								
<12	Reference			Reference			Reference	
>12	1.83 (1.25–2.67)	0.002		3.41 (1.49–7.78)	0.004		NA	NA

*Variables with p<0.1 in Table 2 were included in multivariate unconditional logistic regression analysis. Hosmer-Lemeshow goodness-of-fit test was used to assess the model fit for logistic regression. OR, odd ratio; CI, confidence interval; NA, not available, indicating not included in the final model. †One dependent variable (infection with pandemic [H1N1] 2009 virus) and 5 independent variables (age of index case-patient, type of exposure to index case-patients, age of close contacts, relationships to index case-patients, and exposure duration of close contacts) were included in multivariate analysis. One independent variable (age of index case-patient) was removed in the stepwise regression equation. The goodness-of-fit test suggested that the logistic regression model fitted well (p = 0.631). ‡One dependent variable (infection with pandemic [H1N1] 2009 virus) and 4 independent variables (age of index case-patient, type of exposure to index case-patient, age of close contacts, and exposure duration of close contacts) were included in multivariate analysis. Two independent variables (age of index case-patient and type of exposure to index case-patient) were removed in the stepwise regression equation. The goodness-of-fit test suggested that the logistic regression model fitted well (p = 0.982). §One dependent variable (infection with pandemic [H1N1] 2009 virus) and 5 independent variables (age of index case-patient, type of exposure to index case-patient, age of close contacts, relationships to index case-patient, and exposure duration of close contacts) were included in multivariate analysis. Two independent variables (age of index case-patient and exposure duration of close contacts) were removed in the stepwise regression equation. The goodness-of-fit test suggested the logistic regression model fitted well (p = 0.751). ¶Exposed to symptomatic index case-patients before their illness onset or exposed to index case-patients who had subclinical infections.

## Discussion

We estimated that pandemic (H1N1) 2009 virus was transmitted by 18% of index case-patients to their close contacts and that 2.4% (167 of 7,099) of close contacts we traced were infected. Our data indicate that pandemic (H1N1) 2009 virus has low transmissibility in nonoutbreak settings.

We found that 1 index case-patient transmitted infection to 0.27 close contacts on average, i.e., reproduction number = 0.27. This finding suggests that among those quarantined index case-patients, the number of persons with secondary cases who could be traced through rigorous field investigation was small and far less than the number needed for the sustainable transmission of infectious disease in the population (reproduction number >1). However, the fact that the pandemic eventually spread in Beijing indicates that contact and case tracing were far from complete, especially later in the summer and early fall of 2009. The strict control measures may have worked to some extent at the beginning but were outpaced by local transmission (*16*); the percentage of locally acquired infections ranged from <10% in June 2009 to >80% in September 2009 (data not shown).

In this study, the median number of close contacts per index case-patient per day was 7.0 persons. Although locating and quarantining these close contacts was done quickly, and stringent quarantine measures were used, which hindered implementation of control measures, the real number of close contacts was unknown and probably exceeded this number. Many close contacts were persons met in public places, including public transportation, theaters or cinemas, and shopping malls, and it is nearly impossible to trace all of the contacts. In addition, some persons who had worn PPE during contact with index case-patients were excluded from close contacts management (i.e., they were not quarantined), but because wearing PPE might not protect (or fully protect) against infection, some persons excluded might have become infected. In addition, many persons with mild and asymptomatic cases cannot be detected, but they may transmit the virus. Furthermore, the short generation time of pandemic (H1N1) 2009 shown in this study and in a previous study (*13*) could lead to the rapid accumulation of infection sources and close contacts. This rapid compounding could overwhelm response capacity and would have resulted in compromised effectiveness of containment measures. It should also be mentioned that we did not include persons with cluster or outbreak cases for whom close contacts could not be determined clearly by epidemiologic investigation to examine the basic feature of pandemic (H1N1) 2009 (e.g., generation time), and the reproduction number obtained from our data is an underestimate.

Attack rates of infection differed significantly by contact type. Among household members of index case-patients, the attack rate was the highest, as shown in the multivariate analysis after controlling for age and other factors. The most likely reason for this finding is that household members are more likely to have come into closer contact with index case-patients for a longer period with shorter distance and longer duration. Another possible reason is that household members may have some certain linkage with index cases in genetic susceptibility or living habits that would cause higher predisposition in household members than in other close contacts. This finding is similar to findings in other investigations of respiratory infectious disease (*17*).

Close contacts on flights accounted for the highest proportion of all the close contacts, in part because of how the index cases were detected and the broad definition we used for close contacts. However, the attack rate was much lower than that for other close contacts; 1 index case infected only 0.19 close contacts on flights on average. This finding indicated that the possibility of transmission of pandemic (H1N1) 2009 virus on flights was low, and the yield of tracing and quarantining of close contacts on flights was limited. Tracing contacts of service persons at public places was more difficult than tracing other categories of contacts, and the lowest attack rate (0.2%) was recorded in this category. Despite extensive measures, on average, only 0.01 infected close contacts per index case-patient were identified among service persons. Tracing the contacts of service persons at public places seems far less cost-effective. Criteria for close contacts on flights and those of service persons should be refined with respect to exposure duration and age of those exposed.

Exposure to index case-patients for >12 hours was a significant independent risk factor for infection in flight passenger contacts. This finding suggests that limiting the time of contact with persons with ILI on aircraft can reduce risk for transmission, and a long duration of exposure may be necessary for transmission to occur on aircraft.

Younger close contacts were at higher risk for infection than older ones. The possible reason was that younger persons had much closer contact with index case-patients than did older persons; another reason may be that younger persons were more susceptible to infection with pandemic (H1N1) 2009 virus (*18*). This finding was consistent with findings reported in other studies (*5**,**6*).

No secondary cases were found among close contacts exposed to index case-patients with subclinical infection. The attack rate among close contacts who were exposed to symptomatic index case-patients during their symptomatic phase was much higher than that among those exposed to these case-patients before their illness onset. Exposure to index case-patients during the symptomatic phase was a significant independent risk factor for infection among close contacts. These findings indicate that the infectivity of pandemic (H1N1) 2009 virus was higher after illness onset, and that the infectivity of symptomatic pandemic (H1N1) 2009 case-patients before illness onset was higher than that of persons with subclinical cases, although persons in each group were asymptomatic when in contact with other persons.

In general, the earliest infectious time for pandemic (H1N1) 2009 was considered as 1 day before illness onset (*19*). We found that index case-patients and infected close contacts shed pandemic (H1N1) 2009 virus <1 day before illness onset, which suggests that the infectious period of symptomatic persons with pandemic (H1N1) 2009 might be <1 day before illness onset.

Among close contacts with pandemic (H1N1) 2009, ≈14.4% were asymptomatic. It is noteworthy that specimens from some close contacts tested negative for pandemic (H1N1) 2009 virus before quarantine, but those persons could shed the virus during quarantine without symptoms. Such infection could not be detected, and the proportion of subclinical infection was underestimated. Therefore, we calculated the proportion of subclinical infection by cross-sectional analysis of the subclinical infection of close contacts before quarantine, and we found that ≈17% of case-patients with pandemic (H1N1) 2009 were asymptomatic.

This study has several limitations. We could not find all close contacts of persons with pandemic (H1N1) 2009 and did not know their infection status, so the infection parameters of pandemic (H1N1) 2009 that we found in this study might not be precise, especially for reproduction number, which may be underestimated to some extent. Furthermore, we could not exclude the possibility that the infected close contacts had been infected from another unknown source before quarantine started, which might influence our conclusion to some extent.

In summary, the attack rate among close contacts was low, even among household contacts. Household member and younger age were the major risk factors for infection with pandemic (H1N1) 2009 virus among close contacts. Approximately 17% of cases of pandemic (H1N1) 2009 were asymptomatic.
